# Supplementary material for: Identification of Freezing-Responsive microRNAs and Their Targets in Chinese Jujube by Small RNA and Degradome Sequencing
Source: Int J Mol Sci. 2026 Sep 4;27(17):7912. doi: 10.3390/ijms27177912 (PMC13566890; doi:10.3390/ijms27177912)
Supplement: Supplementary file 1 [file ijms-27-07912-s001.zip › Supplementary Material S2/novel_miR_97.pdf]

Provisional ID : novel_miR_97
Score total : 0.4
Score for star read(s) : -1.3
Score for read counts : 0
Score for mfe : 0.1
Score for randfold : 1.6
Score for cons. seed :
Total read count : 147
Mature read count : 147
Loop read count : 0
Star read count : 0

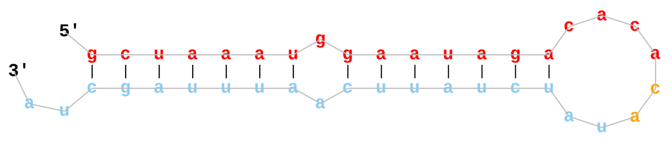

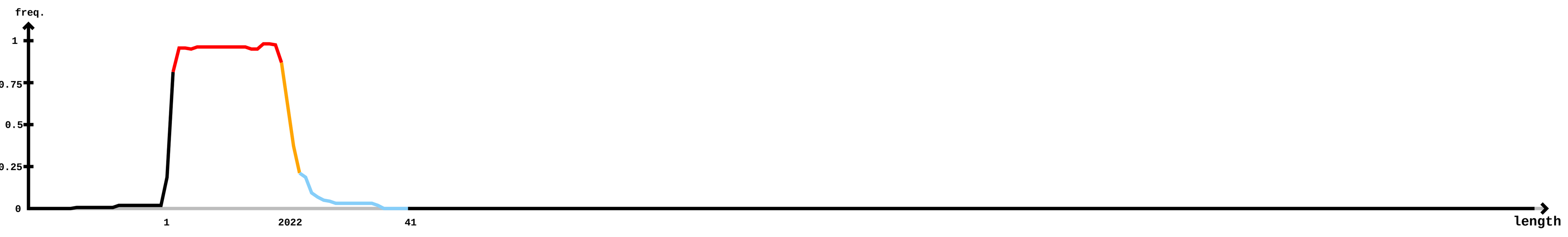

Mature Star

```
5'- agcccaauuugguucucaaaacgcuaaauggaauagacacacauaucuauucaauuuagcuaucaagcacacgcucaaaaauuauuauaaaguguuauucacuuuuuuucuugaccgaugugggauuugaguucauucuauaaauauucuaaacaaauaaauguaaaagauaaaauuauauaauuaaauuucuuuuaaaauguuuguuauuucaaaauuuaucauguauaaauuuuaaugaaauauucau -3'    exp
    ..((((..((((((........(((((((.(((((((........))))))).)))))))......(((....)))............((((((.....)))))).......))))))..))))............................((((((....(((((((......................))))))))...))))))(((((((((((((((.....))))))))...))))))).....    reads   mm   sample
    ......auuugguucucaaaacgcu.................................................................................................................................................................................................................................    1   0   LT-3
    .............ucucaaaacgcuaaauggaaua.......................................................................................................................................................................................................................    2   0   JCK-2
    .....................cgcuaaauggaauagaca...................................................................................................................................................................................................................    1   0   JCK-2
    .....................cgcuaaauggaauagacacU.................................................................................................................................................................................................................    1   1   LT-3
    .....................cgcuaaauggaauagacaca.................................................................................................................................................................................................................    1   0   JCK-2
    .....................cgcuaaauggaauagacaca.................................................................................................................................................................................................................    2   0   LT-3
    .....................cgcuaaauggaauagacaca.................................................................................................................................................................................................................    4   0   JCK-1
    .....................cgcuaaauggaauagacacacU...............................................................................................................................................................................................................    9   1   LCK-2
    .....................cgcuaaauggaauagacacacU...............................................................................................................................................................................................................    6   1   LCK-1
    .....................cgcuaaauggaauagacacacau..............................................................................................................................................................................................................    1   0   JCK-3
    .....................cgcuaaauggaauagacacacau..............................................................................................................................................................................................................    2   0   JCK-2
    ......................gcuaaauggaauagacac..................................................................................................................................................................................................................    1   0   JCK-2
    ......................gcuaaauggaauagacac..................................................................................................................................................................................................................    1   0   LT-1
    ......................gcuaaauggaauagacac..................................................................................................................................................................................................................    3   0   JT-2
    ......................gcuaaauggaauagacac..................................................................................................................................................................................................................    2   0   LT-3
    ......................gcuaaauggaauagacac..................................................................................................................................................................................................................    5   0   LCK-3
    ......................gcuaaauggaauagacac..................................................................................................................................................................................................................    1   0   JCK-3
    ......................gcuaaauggaauagacac..................................................................................................................................................................................................................    3   0   JT-3
    ......................gcuaaauggaCuagacac..................................................................................................................................................................................................................    1   1   JT-2
    ......................gcuaaauggaauagacacU.................................................................................................................................................................................................................    1   1   LT-3
    ......................gcuaaauggaauagacaca.................................................................................................................................................................................................................    16  0   LT-2
    ......................gcuaaauggaauagacacU.................................................................................................................................................................................................................    1   1   LT-1
    ......................gcuaaauggaauagacaca.................................................................................................................................................................................................................    5   0   JCK-2
    ......................gcuaaauggaauagacaca.................................................................................................................................................................................................................    2   0   JCK-3
    ......................gcuaaauggaauagacaca.................................................................................................................................................................................................................    7   0   LT-3
    ......................gcuaaauggaauagacaca.................................................................................................................................................................................................................    9   0   LT-1
    ......................gcuaaauggaauagacaca.................................................................................................................................................................................................................    6   0   JCK-1
    ......................gcuaaauggaauagacaca.................................................................................................................................................................................................................    17  0   LCK-3
    ......................gcuaaGuggaauagacaca.................................................................................................................................................................................................................    1   1   LT-2
    ......................gcuaaauggaauagacacU.................................................................................................................................................................................................................    1   1   JCK-2
    ......................gcuaaauggaauagacacac................................................................................................................................................................................................................    1   0   LT-1
    ......................gcuaaauggaauagacacaA................................................................................................................................................................................................................    1   1   JCK-2
    ......................gcuaaauggaauagacacac................................................................................................................................................................................................................    2   0   JCK-2
    ......................gcuaaauggaauagacacaca...............................................................................................................................................................................................................    1   0   JCK-2
```

```
                      Mature     Star
agcccaauuugguucucaaaacgcuaaauggaauagacacacauaucuauucaauuuagcuaucaagcacacgcucaaaaauuauuauaaaguguuauucacuuuuuucuugaccgauguggauuugaguucauucuauaaauauucuaaacaaauaaauguaaaagauaaaauuauauaauuaaauuucuuuuaaaauguuuguuauuucaaaauuuaucauguauaaauuuuaaugaaauauucau
......................gcuaaauggaauagacacacaua...........................................................................................................................................................................................   1   0   JCK-2
......................gcuaaauggaauagacacacauU...........................................................................................................................................................................................   4   1   LT-2
......................gcuaaauggaauagacacacaua...........................................................................................................................................................................................   1   0   JCK-1
......................gcuaaauggaauagacacacauU...........................................................................................................................................................................................   1   1   LT-1
......................gcuaaauggaauagacacacauCu..........................................................................................................................................................................................   1   1   LT-3
......................gcuaaauggaauagacacacauUu..........................................................................................................................................................................................   1   1   LT-1
......................gcuaaauggaauagacacacauUu..........................................................................................................................................................................................   1   1   LT-2
......................gcuaaauggaauagacacacauCu..........................................................................................................................................................................................   1   1   LT-1
......................gcuaaauggaauagacacacauauU.........................................................................................................................................................................................   1   1   LT-1
......................gcuaaauggaauagacacacauauUu........................................................................................................................................................................................   1   1   JT-3
.......................cuaCauggaauagacaca...............................................................................................................................................................................................   1   1   LT-3
.......................cuaaauggaauagacacU...............................................................................................................................................................................................   1   1   LT-1
.......................cuaaauggaauagacacacU.............................................................................................................................................................................................   1   1   LT-3
.......................cuaaauggaauagacacacU.............................................................................................................................................................................................   9   1   JT-1
.......................cuaaauggaauagacacacau............................................................................................................................................................................................   1   0   LT-1
.......................cuaaauggaauagacacacauU...........................................................................................................................................................................................   1   1   JCK-1
.......................cuaaauggaauagacacacauU...........................................................................................................................................................................................   1   1   JCK-2
.......................cuaaauggaauagacacacauU...........................................................................................................................................................................................   3   1   LT-1
.......................cuaaauggaauagacacacauU...........................................................................................................................................................................................   3   1   LT-3
.......................cuaaauggaauagacacacauauc.........................................................................................................................................................................................   1   0   JCK-2
.......................cuaaauggaauagacacacauauU.........................................................................................................................................................................................   1   1   LT-2
..........................aauggaauagacacacauaucuG.......................................................................................................................................................................................   2   1   JCK-2
.....................................cacacauaucuauucaauu................................................................................................................................................................................   2   0   LT-2
.....................................cacacauaucuauucaauuu...............................................................................................................................................................................   3   0   LT-1
```
